# Clinical Image-Based Dosimetry of Actinium-225 in Targeted Alpha Therapy

**DOI:** 10.3390/cancers18020321

**Published:** 2026-01-20

**Authors:** Kamo Ramonaheng, Kaluzi Banda, Milani Qebetu, Pryaska Goorhoo, Khomotso Legodi, Tshegofatso Masogo, Yashna Seebarruth, Sipho Mdanda, Sandile Sibiya, Yonwaba Mzizi, Cindy Davis, Liani Smith, Honest Ndlovu, Joseph Kabunda, Alex Maes, Christophe Van de Wiele, Akram Al-Ibraheem, Mike Sathekge

**Affiliations:** 1 Nuclear Medicine Research Infrastructure (NuMeRI) NPC, Pretoria 0084, South Africa; 2 Department of Nuclear Medicine, University of Pretoria & Steve Biko Academic Hospital, Pretoria 0001, South Africa; 3 Jawaharlal Nehru Hospital, Rose Belle, Grant Port 51829, Mauritius; 4 Department of Nuclear Medicine, AZ Groeninge, 8500 Kortrijk, Belgium; 5 Department of Diagnostic Sciences, Ghent University, Corneel Heymanslaan 10, 9000 Ghent, Belgium; 6 Department of Nuclear Medicine, King Hussein Cancer Center (KHCC), Amman 11941, Jordan; 7 School of Medicine, University of Jordan, Amman 11942, Jordan

**Keywords:** dosimetry, actinium-225 SPECT imaging, actinium-225 targeted alpha therapy, quantitative SPECT/CT, RBE-weighted dose, dosimetry workflow

## Abstract

Actinium-225 (^225^Ac) has emerged as a pivotal alpha-emitter in modern radiopharmaceutical therapy, offering potent cytotoxicity with the potential for precise tumour targeting. Accurate, patient-specific image-based dosimetry for ^225^Ac is essential to optimize therapeutic efficacy while minimizing radiation-induced toxicity. Establishing a robust dosimetry workflow is particularly challenging due to the complex decay chain, low administered activity, limited count statistics, and the indirect measurement of daughter gamma emissions. Clinical single-photon emission computed tomography/computed tomography protocols with harmonized acquisition parameters, combined with robust volume-of-interest segmentation, artificial intelligence (AI)-driven image processing, and voxel-level analysis, enable reliable time-activity curve generation and absorbed-dose calculation, while reduced mixed-model approaches improve workflow efficiency, reproducibility, and patient-centred implementation. Cadmium zinc telluride-based gamma cameras further enhance quantitative accuracy, enabling rapid whole-body imaging and precise activity measurement, supporting patient-friendly dosimetry. Complementing these advances, the cerium-134/lanthanum-134 positron emission tomography in vivo generator provides a unique theranostic platform to noninvasively monitor ^225^Ac progeny redistribution, evaluate alpha-decay recoil, and study tracer internalization, particularly for internalizing vectors. Together, these technological and methodological innovations establish a mechanistically informed framework for individualized ^225^Ac dosimetry in targeted alpha therapy, supporting optimized treatment planning and precise response assessment. Continued standardization and validation of imaging, reconstruction, and dosimetry workflows will be critical to translate these approaches into reproducible, patient-specific clinical care.

## Simple Summary

Targeted alpha therapy (TAT) using Actinium-225 (^225^Ac) has emerged as a highly promising approach for cancer treatment due to its ability to deliver intense, short-range radiation that effectively eradicates tumour cells while minimizing damage to surrounding healthy tissue. Despite its therapeutic potential, the clinical implementation of ^225^Ac is challenged by the difficulty of accurately assessing radiation distribution at the patient level. These challenges arise from the complex in vivo behaviour of the radionuclide and current limitations in imaging sensitivity, quantification, and standardization. This review summarizes recent advances in imaging methodologies, camera technologies, and artificial intelligence-based image analysis that aim to improve dosimetry and treatment monitoring for ^225^Ac -based therapies. Emphasis is placed on strategies that enhance accuracy, reproducibility, and patient specificity. By consolidating current knowledge and technological developments, this review highlights pathways toward more reliable treatment planning and supports the broader clinical translation and safe adoption of TAT.

## 1. Introduction

### 1.1. Importance of Ac-225 in Modern Radiopharmaceutical Therapy

Prostate-specific membrane antigen (PSMA), highly overexpressed in advanced prostate cancer, has emerged as a cornerstone molecular target for both precision imaging and targeted radionuclide therapy [1,2,3]. β-emitting Lutetium-177 (^177^Lu)-PSMA radioligand therapy has demonstrated efficacy, particularly in taxane-naïve patients [2,4,5,6]; however, a significant proportion of patients (~30–40%) exhibit suboptimal response, and hematologic toxicities remain a concern [3,4]. These limitations have driven interest in targeted alpha therapy (TAT), which exploits the unique properties of alpha particles—high linear energy transfer (LET) ~80 keV/μm and a very short range (40–100 μm)—causing dense, localized, and largely irreparable deoxyribonucleic acid damage in tumour cells while minimizing effects on surrounding healthy tissue [7]. In contrast, beta particles deposit lower LET (~0.2 keV/μm) over much longer distances (0.05–12 mm), producing more diffuse energy deposition [8].

Actinium-225 (^225^Ac) is a potent alpha-emitting radionuclide that can be incorporated into PSMA-targeting vectors such as [^225^Ac]Ac-PSMA-617, [^225^Ac]Ac-PSMA-I&T, and [^225^Ac]Ac-J591, where it induces strong and, in some cases, sustained antitumour responses [9,10,11,12,13,14,15]. The development of radiolabelling approaches employing ethylene diamine tetraacetic acid, 1,4,7,10,13,16-hexaacetic acid, diethylenetriaminepentaacetic acid,1,4,7,10-tetraazacyclododecane-1,4,7,10-tetrapropionic acid, 1,4,7,10-tetraazacyclododecane-1,4,7,10-tetraacetic acid, and macrocyclic picolinic acid chelators has facilitated the clinical translation of ^225^Ac-based constructs [16,17,18,19]. Additional investigational agents, including [^225^Ac]Ac-humanized monoclonal antibody (hu11B6), [^225^Ac]Ac-CD46-targeting human antibody (YS5), and [^225^Ac]Ac-(S)-Ibuprofen-diaminobutyric acid (SibuDAB), are being explored for targeted delivery to human kallikrein peptidase 2, membrane cofactor protein, and PSMA, respectively [5,20,21,22]. Clinical studies of [^225^Ac]Ac-PSMA radioligand therapy have demonstrated significant PSA reductions, durable remissions, and acceptable toxicity profiles characterized primarily by dose-dependent xerostomia, with severe hematologic or renal toxicity reported only rarely [5,11,23,24].

Despite promising outcomes, TAT development remains constrained by ^225^Ac production challenges, daughter nuclide redistribution, and limited imaging capabilities. Given the high radiobiological potency of alpha particles and the growing clinical adoption of [^225^Ac]Ac-PSMA therapy [15], the development of accurate image-based dosimetry is critical. Accurate dosimetry enables patient-specific therapy optimization, reliable dose–response assessment, and minimization of toxicity to organs at risk. It also supports safe, evidence-based activity selection, ultimately facilitating standardized and effective clinical implementation of alpha-emitter radioligand therapy. The therapeutic efficacy of ^225^Ac is intricately linked to accurate dose delivery, making robust dosimetry essential for optimizing treatment outcomes.

Traditional dosimetry methods often rely on physical measurements and complex calculations that may not accurately reflect the biological effects of radiation on tissues.

This review provides a comprehensive analysis of current image-based dosimetry methodologies for ^225^Ac targeted alpha therapy, with a particular emphasis on ^225^Ac clinical imaging protocols for dosimetry based on decay emission and the clinical quantitative imaging and dosimetry workflow. It reviews optimized single-photon emission computed tomography/computed tomography (SPECT/CT) acquisition strategies, harmonized activity quantification and reconstruction approaches, and advanced dosimetry workflows to support accurate absorbed-dose calculation. Practical considerations for patient-centred dosimetry, including the use of state-of-the-art imaging systems to reduce scan times and enhance feasibility, are discussed. The article outlines strategies to improve safety assessment, individualized treatment planning, and therapeutic response evaluation, highlighting the transformative potential of image-based dosimetry in ^225^Ac radiopharmaceutical therapy.

### 1.2. Overview of Actinium-225 Production Routes

^225^Ac was initially discovered in 1947 through independent work by researchers at Argonne National Laboratory and a Canadian group led by A. C. English [25,26]. The potential application of ^225^Ac, along with ^213^Bi, for radioimmunotherapy was not proposed until 1993, when Geerlings et al. first suggested their use as therapeutic agents [27].

**Figure 1:**
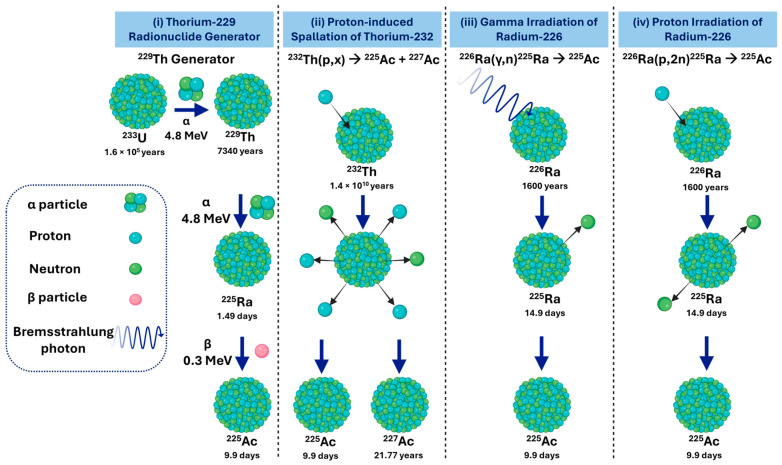
Overview of actinium-225 production pathways, including (**i**) thorium-229 radionuclide generator, (**ii**) proton-induced spallation of thorium-232, (**iii**) gamma irradiation of radium-226, and (**iv**) proton irradiation of radium-226. Created with BioRender.com (N/A Student Plan, Science Suite Inc., Toronto, Ontario, ON, Canada). Adapted from [49].

Current major producers of ^225^Ac [28,29,30,31] include JRC Karlsruhe in Germany, supplying through the PRISMAP Project alongside CERN in Geneva, Oak Ridge National Laboratory (ORNL; Oak Ridge, TN, USA), which sources material via the National Isotope Development Center funded by the DOE Isotope Program [32,33,34,35,36], and the Institute of Physics and Power Engineering (IPPE; Obninsk, Russia) [37]. In the United States, additional production is carried out by Brookhaven National Laboratory (BNL; Upton, NY, USA), which began independent ^225^Ac production in 2022, and Los Alamos National Laboratory (LANL; Los Alamos, NM, USA), which provides irradiation support. Emerging US suppliers include TerraPower (Bellevue, WA, USA) [38], Ionetix (Lansing, MI, USA) [39], and NorthStar Medical Radioisotopes (Beloit, WI, USA) [40], with the ^225^Ac Tri-Lab Effort (ORNL, BNL, LANL) coordinating DOE resources to address supply gaps [41]. In Canada, ^225^Ac is produced at TRIUMF (Vancouver, Canada) [42,43], Canadian Nuclear Laboratories (Chalk River, Canada) [44], and commercially by BWXT Medical Ltd. (Ottawa, Canada). Additional production occurs in Asia at the Institute of Material Research, Tohoku University (Sendai, Japan) [45,46] and the China Isotope and Radiation Corporation (Beijing, China) [47]. In Russia, the Institute for Nuclear Research of the Russian Academy of Sciences (INR RAS; Moscow, Russia) also contributes to the supply [48]. Ongoing efforts to expand legacy DOE thorium-229 (^229^Th) stocks are expected to improve availability; however, the overall ^225^Ac supply remains constrained and insufficient to meet long-term clinical demand. Clinical and research use of ^225^Ac relies on four production pathways (Figure 1), each with distinct implications for availability, purity, and scalability [28,29].

i. Actinium-225 from a Thorium-229 Radionuclide Generator

The decay of ^229^Th (T_1/2_ = 7917 years) remains the historic and clinically established route for producing high-purity ^225^Ac [28]. Originating from aged uranium-233 (Figure 1i), ^229^Th is processed through a series of radiochemical separation steps, including anion and cation exchange, as well as extraction chromatography in nitric acid, to isolate carrier-free, clinical-grade ^225^Ac with recovery yields typically exceeding 95% [29,50]. This generator system also provides access to bismuth-213 (^213^Bi), enabling both TAT and theranostic applications.

Although the ^229^Th/^225^Ac generator produces exceptionally high-quality material, production capacity is inherently limited by the long half-life of the parent isotope and the restricted global inventory of ^229^Th.

ii. Actinium-225 via Proton-Induced Spallation of Thorium-232

High-energy proton irradiation of Thorium-232 (^232^Th) targets via the ^232^Th(p,x)^225^Ac spallation route (Figure 1ii) remains the most technically mature and scalable accelerator-based method for large-volume ^225^Ac production, and it is currently implemented at the major U.S. Tri-Lab facilities, ORNL, LANL, and BNL [28]. In this approach, spallation of ^232^Th generates substantial quantities of ^225^Ac together with a broad distribution of fission and spallation products. A persistent challenge is the unavoidable co-production of long-lived actinium-227 (^227^Ac) (T_1/2_ = 21.8 y), typically present at 0.1–0.2% of the ^225^Ac activity at end-of-bombardment. ^227^Ac is chemically inseparable from ^225^Ac on clinically relevant timescales, therefore, its presence introduces regulatory, radiological safety, and long-term waste-management considerations despite early dosimetry studies indicating limited clinical impact at these impurity levels [51].

To mitigate ^227^Ac contamination, the ^232^Th spallation route can be redirected toward the production of ^225^Ra, which subsequently decays to generator-grade ^225^Ac with high isotopic purity. However, this generator-based alternative yields significantly smaller quantities of ^225^Ac compared with direct spallation production [52]. As such, while spallation remains the only method currently capable of meeting future multi-Curie-scale demand, it involves a trade-off between production volume and isotopic purity.

iii. Actinium-225 via Gamma Irradiation of Radium-226

Photon-induced production of ^225^Ac via the radium-226 (^226^Ra) (γ,n)^225^Ra reaction (Figure 1iii) provides an indirect route in which ^225^Ra, generated through gamma-induced neutron emission, subsequently decays to yield ^225^Ac. While theoretically capable of yielding large quantities of ^225^Ac, this route has not yet been demonstrated at scales suitable for clinical application. Current limitations stem from the scarcity of sufficiently intense gamma sources and the absence of proof-of-principle studies showing production of high-activity batches. As such, although scientifically viable, the gamma irradiation pathway remains at an exploratory stage compared with the more established proton-irradiation method.

iv. Actinium-225 via Proton Irradiation of Radium-226

Medium-energy proton irradiation of ^226^Ra via the ^226^Ra(p,2n)^225^Ac reaction (Figure 1iv) is the most mature accelerator-based production route for high-purity ^225^Ac. Irradiation at approximately 15–16 MeV maximises the formation of ^225^Ac while minimising co-production of short-lived impurities such as actinium-226 (^226^Ac) (T_1/2_ = 29 h) and actinium-224 (^224^Ac) (T_1/2_ = 2.9 h), which decay during the standard post-irradiation cooling period. A 24 h irradiation of ~50 mg of ^226^Ra at 100 μA typically yields around 5 GBq of ^225^Ac, sufficient for several hundred clinical doses, demonstrating both efficiency and scalability [28,53]. Challenges include safe handling of ^226^Ra and its gaseous daughter radon-222 (^222^Rn), target fabrication and encapsulation, and the need for specialised radiochemical and cyclotron infrastructure. Alternative accelerator-based production routes, such as deuteron-induced formation of ^225^Ac via the ^226^Ra(d,3n)^225^Ac reaction, or photonuclear generation of radium-225 (^225^Ra) through the ^226^Ra(γ,n)^225^Ra pathway, remain scientifically promising but require further cross-section benchmarking, target optimization, and reliable access to high-intensity, high-energy beams.

Table 1 summarizes the principal production routes for ^225^Ac, comparing yield, isotopic purity, radiological and dosimetry characteristics, and their suitability for clinical and theranostic applications. This overview provides a concise reference for production methods based on clinical demand, and radiation safety considerations.

The ^225^Ac production route via ^229^Th generators, ^232^Th spallation, or ^226^Ra irradiation determines radionuclide purity, specific activity, and co-produced isotopes such as ^227^Ac, all of which directly impact SPECT/CT-based image quantification. Co-emitted gamma photons from impurities influence counting statistics and require careful gamma window selection, while variations in specific activity affect signal intensity and optimal imaging time points. Additionally, waste streams containing these radionuclide impurities must be carefully characterized, decay-stored, and handled in compliance with regulatory requirements, as residual long-lived isotopes not only pose radiological hazards but can also compromise the accuracy and reproducibility of quantitative imaging and patient-specific dosimetry.

### 1.3. Waste Management in Actinium-225 Radiopharmaceutical Production

Effective waste management in ^225^Ac radiopharmaceutical production requires a comprehensive, continuously implemented strategy encompassing waste minimization, accurate radionuclide characterization, appropriate conditioning and packaging, secure interim storage, and compliant final disposal. Although α-emitting radionuclides pose minimal external exposure risk to radiation workers due to their limited penetration, accidental internal uptake (via ingestion, inhalation, or wounds) can result in significant dose consequences. Regulatory frameworks and licensing conditions therefore impose strict controls to prevent internal contamination and ensure that all handling, storage, and disposal procedures meet radiological protection requirements [54,55].

Waste management begins at the point of radionuclide receipt and must be informed by complete documentation from the supplier [54]. This is particularly critical because the radionuclide composition of waste varies substantially with the production route. ^229^Th generator, derived ^225^Ac is essentially free of long-lived contaminants such as ^227^Ac, whereas accelerator-based production routes, such as ^232^Th spallation or ^226^Ra proton irradiation, may introduce long-lived nuclides including ^226^Ra, ^229^Th, ^225^Ac residuals, and potentially trace fractions of ^227^Ac. For all ^225^Ac sources other than ^229^Th generators, explicit reporting of ^227^Ac content is essential, as many jurisdictions enforce very low exemption limits for this nuclide [55]. These strict limits can pose significant challenges in classifying and disposing of waste from high-yield accelerator production.

^226^Ra-based production routes (both proton- and gamma-induced) may generate waste streams containing parent radium and its decay products, including gaseous ^222^Rn, necessitating specialized containment and ventilation protocols. Thorium-containing residues arising from ^232^Th spallation or from generator materials may require transfer to licensed long-term storage or disposal facilities, depending on regional regulations.

Local radiation protection authorities should be consulted to determine whether consumables and components used during production, such as synthesis cartridges, tubing, shielding, or vials, may be decay-stored until activities fall below clearance thresholds. Decay-in-storage can significantly reduce ^225^Ac activity: storage of contaminated material for 100 days may reduce activity by a factor of 1000, while storage for one year can reduce activity by more than 1 million [54]. If regulations require that all material be deposited at a licensed depository, the availability of a satisfactory deposit must be verified. In some cases, decay-in-storage may permit reclassification of materials as non-radioactive waste; however, this is generally not applicable for items contaminated with long-lived radionuclides such as ^229^Th, ^226^Ra, or ^227^Ac.

Collectively, the diversity of ^225^Ac production pathways necessitates waste-management protocols tailored to the specific isotopic signature of each source. A robust, well-documented, and regulatory-aligned waste program is therefore central to ensuring radiation safety, environmental protection, and sustainability as clinical use of ^225^Ac continues to expand.

### 1.4. Decay Properties of Actinium-225 for Theranostics and Dosimetry Applications

**Figure 2:**
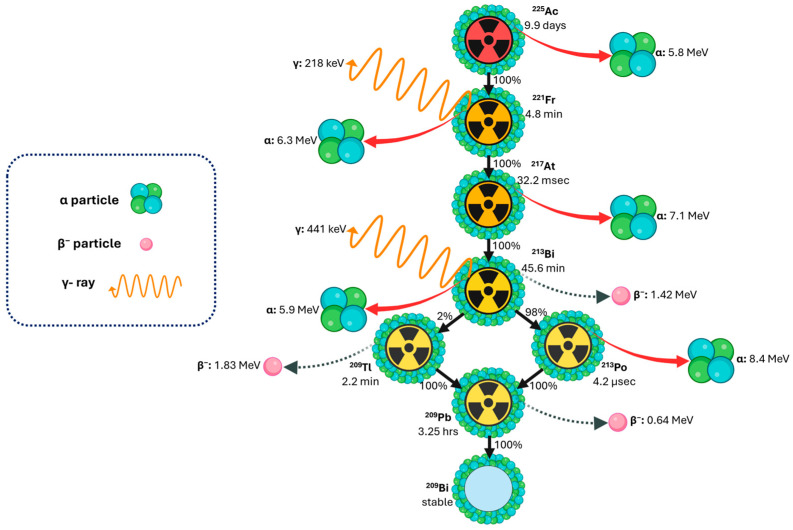
Decay scheme of actinium-225, showing key emissions and daughter radionuclides relevant for theranostics and dosimetry applications. Created with BioRender.com. Adapted from [56].

^225^Ac decays through a cascade (Figure 2) of six short-lived daughter radionuclides, with a physical half-life of 9.92 days, ultimately reaching stable ^209^Bi [57]. This decay chain results in the emission of four net α-particles, two β-particles, and two γ-emissions, with the α-particles exhibiting short path lengths (40–100 μm), maximizing localized cytotoxicity while minimizing off-target toxicity [52,58]. The decay of ^225^Ac releases recoil energies of 100–200 keV, sufficient to disrupt chemical bonds and liberate daughter isotopes from chelators [59]. This phenomenon poses a major challenge for TAT, as unbound daughters can migrate and increase dose to non-target tissues unless internalization and intracellular retention are achieved. Consequently, understanding the in vivo behaviour of ^225^Ac daughters is critical for accurate dosimetry and toxicity assessment [57,58].

The principal α-emitting progeny of ^225^Ac include francium-221 (^221^Fr) (E_α_ ~6.3 MeV, T_1/2_ = 4.8 min), astatine-217 (^217^At) (E_α_ ~7.1 MeV, T_1/2_ = 33 ms), and ^213^Bi (E_α_ ~5.8 MeV, T_1/2_ = 45.6 min), which proceed through branching decay pathways involving polonium-213 (^213^Po) and thallium-209 ^(209^Tl), ultimately yielding stable ^209^Bi. Imaging and quantitative pharmacokinetic assessment are facilitated by two diagnostically useful γ-emissions: 218 keV from ^221^Fr (11.4% abundance) and 440 keV from ^213^Bi (25.9% abundance). The extremely short physical half-life of ^221^Fr substantially limits its ability to migrate away from the parent decay site, whereas ^213^Bi, with a longer physical half-life and greater biochemical mobility, can undergo measurable redistribution, thereby contributing to off-target absorbed-dose deposition in normal tissues [7,19,26,58].

Although the short half-life of ^221^Fr limits its migration and the longer-lived ^213^Bi may redistribute, contributing to off-target absorbed dose in normal tissues, the therapeutic efficacy of ^225^Ac is highlighted by the clinical success of radium-223 dichloride (Xofigo) in the treatment of bone metastases in castration-resistant prostate cancer [19]. Unlike ^223^Ra, the Ac^3+^ ion exhibits a large ionic radius (112 pm) and low charge-to-radius ratio, resulting in weak electrostatic interactions with conventional chelators. Developing thermodynamically stable and kinetically inert complexes for ^225^Ac remains an active area of research [59]. To date, chelator development has been limited by the restricted availability of ^225^Ac, which constrains preclinical and clinical studies [19].

### 1.5. Daughter Redistribution, Recoil Effects, and Implications for Dosimetry

**Figure 3:**
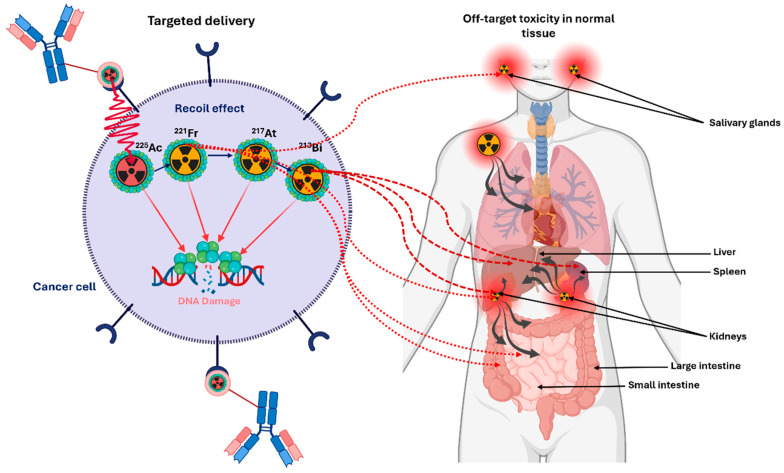
Schematic of actinium-225 targeted alpha therapy showing chelated vector delivery to the tumour, α-decay daughter production, recoil-induced redistribution of daughters, and potential off-target dose to normal tissues. Created with BioRender.com.

The nuclear recoil energy released, typically 100–200 keV, far exceeds chemical bond energies, making bond rupture unavoidable and ensuring that daughter nuclides dissociate from the parent radiopharmaceutical [60]. Because recoil is intrinsic to the physics of α-decay, daughter release is an expected phenomenon that must be incorporated directly into dosimetry models rather than treated as a correctable chemical limitation. Following dissociation, daughter behaviour (Figure 3) is governed by ballistic recoil, diffusion, and organ-specific biological transport, which may produce unintended absorbed dose to healthy tissues [61]. The following three mitigation strategies have been described in the literature, designed to limit systemic redistribution of α-emitting daughters, although they cannot prevent the intrinsic nuclear recoil process.

(i) Encapsulation in nanocarriers, (ii) rapid intracellular internalization, and (iii) local administration, such as intratumoural injection [59].

This issue carries particular dosimetric importance for the ^225^Ac decay chain. The recoil from ^225^Ac α-decay disrupts the chelator–metal complex, releasing progeny whose chemical properties differ from the parent and may display reduced chelator affinity [59,62]. Among these, ^213^Bi (T_1/2_ = 45.6 min) is noteworthy: its comparatively long physical half-life enables redistribution over physiologically relevant timescales, unless the vector is internalized and retained within tumour cells. Consequently, later α-emissions may occur at off-target sites, complicating absorbed-dose estimates.

**Figure 4:**
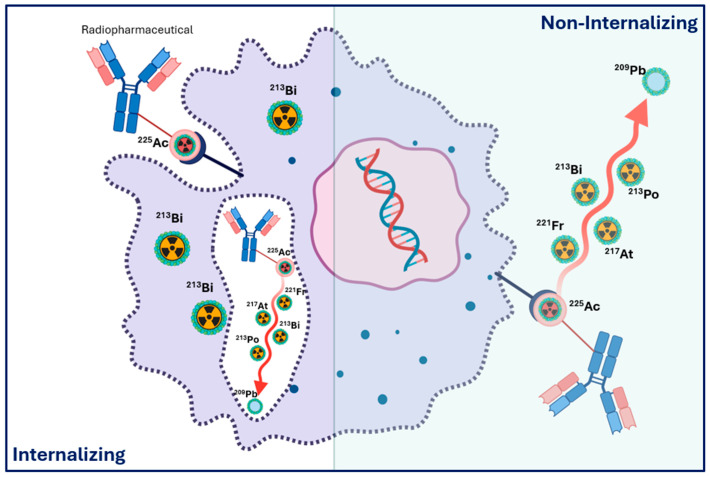
An illustration of how internalizing versus non-internalizing radiotracers affect ^225^Ac progeny redistribution, with photon emissions used for imaging and activity quantification in dosimetry indicated. Created with BioRender.com. Adapted from [63].

The ^134^Ce/^134^La theranostic pair provides a means to indirectly investigate, and potentially mitigate, the effects of α-decay recoil on TAT by serving as a diagnostic surrogate for ^225^Ac [63]. When internalizing vectors such as PSMA-617 are labelled with ^134^Ce, the in vivo distribution of the PET-emitting daughter ^134^La can be monitored, offering insight into progeny retention versus meandering following decay. Although ^134^Ce PET underestimates tumour uptake for slow-internalizing tracers (Figure 4), due to redistribution of ^134^La, in this work, Bauer et al. [63] demonstrated that the ^134^Ce/^134^La pair supports noninvasive assessment of progeny redistribution relevant to ^225^Ac.

Preclinical investigations demonstrate that freely circulating ^213^Bi accumulates in the kidneys [62,64]. In biodistribution studies of ^225^Ac-huM195, Schwartz et al. [64] reported effective half-lives of 6.7 days for ^225^Ac and 4.6 days for non-equilibrium circulating ^213^Bi, emphasizing that progeny pharmacokinetics must be treated as distinct, non-local equilibrium processes. As a result, daughter-specific time-activity curves (TACs) are required for accurate dosimetry. In certain studies, biological clearance curves derived from ^177^Lu-PSMA have been employed as surrogates for ^225^Ac, however, this approach may overestimate renal absorbed dose, as portions of the ^225^Ac decay chain can redistribute outside the kidneys [62,64]. Accurate ^225^Ac dosimetry necessitates serial, progeny-specific quantitative imaging to resolve the dynamic biodistribution of each daughter. Comprehensive dose assessment must integrate recoil-mediated daughter release, differential progeny pharmacokinetics, organ-specific redistribution, and the non-equilibrium kinetics of the decay chain, all of which critically shape the spatial pattern of absorbed dose.

Routine quantitative imaging of ^225^Ac remains technically challenging due to the low administered activities (~4 to 8 MBq) [11,65], which result in poor photon statistics per projection. To compensate, longer acquisition times of up to one hour per bed position may be required, imposing patient discomfort and limiting clinical throughput [64]. Low signal-to-noise ratios reduce both spatial and temporal resolution, complicating accurate delineation of organs and lesions, rapidly evolving daughter-specific biodistribution influenced by recoil and variable pharmacokinetics, and generating comprehensive TACs required for accurate dosimetry. Despite these limitations, modern single-photon emission computed tomography (SPECT) systems and reconstruction techniques increasingly support clinically meaningful quantification, although substantial methodological refinement is still required.

Post-therapy quantitative SPECT/CT enables evaluation of ^225^Ac pharmacokinetics, but accurate image-based dosimetry is complicated by α-decay recoil, which induces disequilibrium within the decay chain. The commonly used photopeaks, 218 keV from ^221^Fr and 440 keV from ^213^Bi, originate from daughter radionuclides that may have redistributed from the parent ^225^Ac decay site by the time of imaging. As a result, the detected photon emissions represent a superposition of spatially and kinetically independent distributions, rather than a single coherent activity map, complicating quantitative reconstruction and organ- or lesion-level dosimetry [7,16,19,52,59]

^221^Fr and ^213^Bi exhibit distinct biological clearance rates and residence times, therefore their photopeak signals cannot be combined under assumptions of spatial coincidence. This decay-chain disequilibrium generates radionuclide-specific partial-volume effects (PVEs), energy-dependent scatter variations, and potential misregistration errors when conventional reconstruction algorithms assume a uniform source distribution. Consequently, accurate quantitative SPECT/CT of ^225^Ac necessitates energy-resolved reconstruction and daughter-specific calibration factors to reliably extract TACs and compute organ- and lesion-level absorbed doses [7,16,19,52,59]. These challenges emphasize the critical importance of implementing a robust, clinically applicable image-based dosimetry workflow for ^225^Ac, which integrates sequential imaging, progeny-specific analysis, and rigorous calibration to support accurate treatment planning and radiobiological assessment.

### 1.6. Clinical Quantitative Imaging and Dosimetry Workflow for Actinium-225

**Figure 5:**
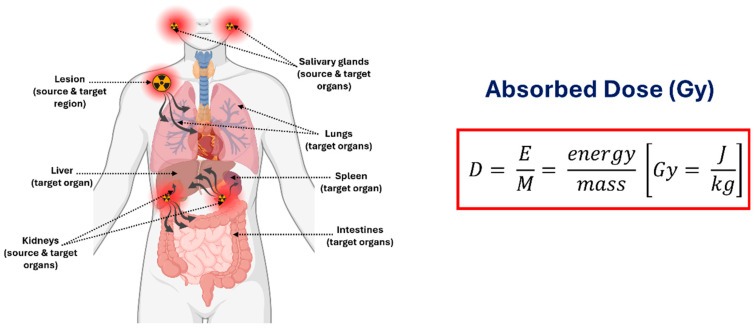
Schematic illustration of absorbed-dose concepts in ^225^Ac therapy, indicating energy deposition (Gy) per unit mass in target regions, including normal organ tumour lesions, arising from source-region activity distributions.

**Figure 6:**
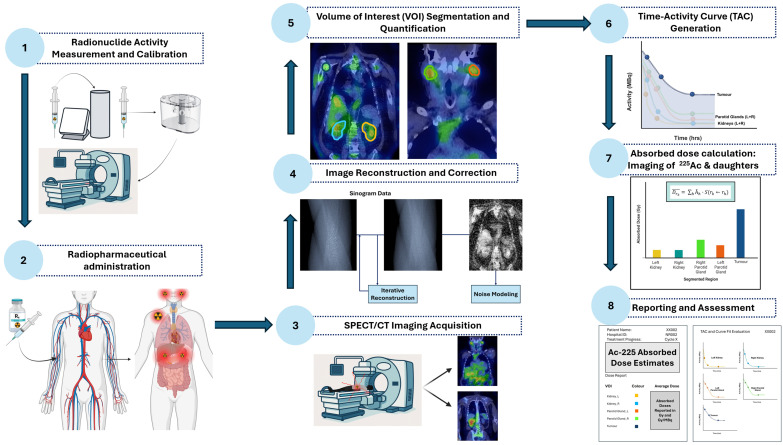
Clinical image-based dosimetry workflow for ^225^Ac, from imaging and activity quantification to dose calculation and reporting.

The absorbed dose (Gy) represents the energy deposited per unit mass (Figure 5) in a defined target region, which may include normal organs (e.g., kidneys, salivary glands) or tumour lesions. Accurate dose calculation further requires characterizing the source regions responsible for radiation emission, including organs that concentrate the parent radionuclide (e.g., liver, spleen) as well as tissues that may transiently accumulate daughter nuclides due to recoil-mediated redistribution. The delivered absorbed dose ultimately depends on several interrelated factors, including administered activity, physical and chemical properties of the radiopharmaceutical, the geometric relationship between source and target compartments, and the biokinetics and biodistribution governing uptake, retention, and biological clearance in both normal organs and tumours, as emphasized in the European Association of Nuclear Medicine Focus meeting [66]. For theranostic applications, the workflow (Figure 6) for image-based dosimetry of ^225^Ac may proceed as follows.

**Core Steps in ^225^Ac Image-Based Dosimetry:**

1. Radionuclide Activity Measurement and Calibration—Measure administered activity using a calibrated dose calibrator.Perform gamma camera cross-calibration with phantoms to convert image counts to units of activity.
2. Radiopharmaceutical Administration—Deliver the radiopharmaceutical intravenously and account for residual activity to determine the net administered dose.
3. SPECT/CT Imaging Acquisition—Acquire patient imaging at clinically relevant time points, targeting primary photopeaks of ^225^Ac progeny.
4. Image Reconstruction and Correction—Apply iterative reconstruction with corrections for attenuation, scatter, collimator-detector response (CDR) modelling, and camera-specific calibration factors.
5. Volume-of-Interest Segmentation—Delineate organs and tumours using computed tomography (CT), AI-assisted, or threshold-based methods; propagate volumes of interest (VOIs) across time points to quantify activity while minimizing PVEs.
6. Time-Activity Curve Generation—Extract activity in each volume of interest (VOI) over time to generate TACs, fit kinetic models, and compute time-integrated activity (TIA) and time-integrated activity coefficients (TIACs).
7. Absorbed Dose Calculation—Generate voxel-level dose maps by convolving voxel S-value (VSV) kernels with TIA distributions. Compute organ- and tumour-level doses and apply relative biological effectiveness (RBE) weighting for α-particle effects.
8. Reporting and Biological Assessment—Present absorbed doses with uncertainties, dose-volume metrics, and biological interpretation. Evaluate organs at risk to guide safe treatment cycles and absorbed tumour doses to incorporate patient-specific radiobiological considerations.

These steps are elaborated in detail below.

#### 1.6.1. Radionuclide Activity Measurement and Calibration

The administered activity of the ^225^Ac-labeled radiopharmaceutical is measured using a certified dose calibrator, specifically calibrated for ^225^Ac and traceable to a reference standard, providing precise and reliable input for the dosimetry. Cross-calibration between the gamma camera and dose calibrator is typically performed using phantoms with uniformly filled cylindrical activity, hot solid spheres, or hot rod inserts, allowing derivation of robust calibration factors (CFs) for the gamma camera [67]. The phantom acquisition protocol should replicate the patient imaging protocol to improve the accuracy of the converted reconstructed image counts into activity concentrations, while accounting for physical decay and the inherently low administered activities (commonly 4–8 MBq) [65], which pose significant challenges for quantitative SPECT/CT images. Employing a calibration protocol identical to that of patient studies mitigates reconstruction-related inaccuracies and enhances the robustness and reproducibility of the quantitative activity measurements.

#### 1.6.2. Radiopharmaceutical Administration

Following intravenous administration, all residual activity in the emptied syringe and any associated administration components is measured to determine the net administered activity, ensuring precise quantification of the radiopharmaceutical delivered to the patient.

#### 1.6.3. SPECT/CT Imaging Acquisition

Recent advances in low-count quantitative SPECT/CT enable imaging of ^225^Ac via the 440 keV gamma emission of ^213^Bi, facilitating early clinical validation of patient-specific dosimetry [68,69]. Post-therapy imaging is typically performed at clinically relevant time points, ranging from 4 h to 168 h post-injection, to capture the evolving spatial and temporal distribution of ^225^Ac and its daughter radionuclides (^213^Bi, ^221^Fr) [70,71,72]. Imaging is performed using high-energy (HE) collimators (generally high-energy general-purpose (HEGP) with 3/8″ sodium iodide doped with thallium crystals), targeting the principal photopeaks of the decay chain: 440 keV (^213^Bi), 218 keV (^221^Fr), and optionally 78 keV or 92 keV X-ray emissions. Low-dose CT is employed for attenuation correction to ensure accurate quantification of activity distributions. Acquisition protocols for quantitative SPECT/CT imaging used in dosimetry, including scan times, projections, and energy windows, are described in detail in the subsequent section. Accurate ^225^Ac dosimetry typically relies on multi-time-point imaging, with three time points sufficient to capture temporal activity distributions during the initial therapy cycle [70,71]. For subsequent cycles, a single time-point acquisition can provide a reasonable compromise between quantitative accuracy and patient burden. Hybrid approaches, in which a single SPECT/CT acquisition is complemented by multiple planar scans at additional time points, offer a practical strategy to maintain dosimetry reliability while accommodating clinical constraints. Establishing such minimum clinically acceptable imaging schedules is particularly relevant for centres with limited scanner availability or patients with restricted tolerance for repeated imaging.

#### 1.6.4. Image Reconstruction and Correction

SPECT images are reconstructed using iterative or otherwise validated algorithms to maximize both image quality and quantitative accuracy [73]. Compared with planar imaging, SPECT offers superior quantitative accuracy due to reduced overlap of accumulating activity in the projection line and the ability to incorporate image-degrading corrections directly within the iterative reconstruction process. Corrections are applied for photon attenuation (derived from low-dose CT), scatter, and resolution effects, including septal penetration and scatter. The above-mentioned phantom-based CFs are used to convert voxel counts into activity concentrations. For quantitative SPECT/CT analysis, reconstructed images corresponding to one or more primary energy windows (e.g., 218 keV and/or 440 keV photopeaks) may optionally be summed post-reconstruction to generate a composite activity distribution for dosimetry evaluation, depending on the specific photopeak(s) of interest [69].

#### 1.6.5. Volume of Interest Segmentation for Activity Quantification

VOIs are delineated for organs at risk, such as the kidneys and salivary glands, as well as for tumour lesions on co-registered SPECT/CT images. Organ and tumour VOIs can be segmented using CT-based segmentation, AI-based automated methods or threshold-based isocontours, with manual refinement applied as necessary to optimize accuracy [74,75]. For improved accuracy in activity quantification and efficacy assessment, both tumour volume definition and tumour dose interpretation are highly sensitive to the choice of imaging modality [76]. The substantial variations in tumour doses underscore the critical need for standardized protocols for multimodality tumour segmentation in targeted radionuclide therapy (TRT) dosimetry. The partial volume effect (PVE) remains a major source of uncertainty when estimating in vivo radionuclide uptake, particularly in small structures. Accurate absorbed dose estimation in radionuclide therapy is therefore challenging until robust and widely applicable PVE correction strategies are established [77]. Performance characteristics, such as limited spatial resolution, the choice of segmentation threshold, and the presence of small tumours near the system’s resolution limit all contribute to susceptibility to PVE, further complicating reliable tumour activity quantification. VOIs may be propagated across serial imaging time points, with adjustments to accommodate anatomical or positional changes. Activity within each VOI is quantified over time to generate TACs, which form the basis for accurate organ- and lesion-specific dosimetry calculations. This workflow allows for the reliable estimation of absorbed doses from ^225^Ac and its decay daughters, integrating spatial and temporal variations in distribution to support patient-specific, image-based dosimetry.

#### 1.6.6. Time–Activity Curve Generation

VOIs corresponding to source regions (e.g., tumours) or target organs (e.g., kidneys, salivary glands), are quantified for total activity at multiple imaging time points to generate model-derived TACs that characterize radiopharmaceutical kinetics. Mono-exponential models are typically fitted to these TACs to estimate effective half-lives, accounting for both physical decay and biological clearance, while multi-exponential fitting is feasible when at least four time points are available. Decay correction is applied to ensure accurate representation of the differing half-lives of ^225^Ac and its daughters. The resulting effective half-lives are then applied on a voxel-wise basis, and the model-derived activity–time functions are integrated, either VOI-wise or voxel-wise, to obtain the TIA for each source or target region [70,78]. The time-integrated activity coefficient (TIAC) is subsequently obtained by normalizing the TIA to the administered activity of ^225^Ac or expressing it as a fraction of the administered activity. The contribution of variability arising from curve fitting and numerical integration is generally small for healthy organs, and can be further minimized by following best-practice recommendations, including appropriate function selection, pharmacokinetic modelling, and systematic sanity checks [78].

#### 1.6.7. Absorbed Dose Calculation

VSV kernels represent the mean absorbed dose delivered to a target voxel per radioactive decay in a source voxel within a homogeneous medium. By convolving these precomputed kernels with the spatial distribution of time-integrated activity from imaging, voxel-level absorbed dose maps can be efficiently generated. Absorbed dose maps are typically generated using VSV kernels for ^225^Ac [79,80]. Organ- and tumour-specific doses are then calculated using established dosimetric frameworks such as the MIRD formalism, incorporating localized energy deposition from both alpha and beta emissions. To account for the increased biological effectiveness of alpha particles, absorbed doses are commonly weighted using an RBE (see Section 1.8.7 for further detail) factor of approximately five. Self-irradiation may be considered for all compartments during dose computation. Dose attribution can be derived from several imaging surrogates, including ^213^Bi SPECT or ^221^Fr SPECT as proxies for ^225^Ac and its daughters, or by combining both isotopes to capture contributions from short-lived progeny such as ^217^At, ^221^Fr, and ^213^Bi. Corresponding S-values required for these calculations can be obtained from established open-access dosimetry resources using pre-simulated VSV kernels [81].

#### 1.6.8. Reporting and Biological Assessment

TACs and TIACs may be derived from the primary daughter photopeaks, such as ^221^Fr and ^213^Bi, to evaluate the degree of daughter retention within the delivery vector compared with redistribution to other organs, including the kidneys [71]. This assessment provides important insight into whether multiple α-particle emissions occur within the tumour, thereby supporting the therapeutic potential of ^225^Ac and contributing to a favourable tumour-to-background profile. Absorbed doses calculated from combined daughter photopeaks can be compared with those obtained from individual peaks to determine whether combining daughter photopeaks improves the signal-to-noise ratio or yields more robust, stable dose estimates. Furthermore, absorbed-dose estimates may offer valuable guidance on the number of treatment cycles that can be safely administered by benchmarking against established organ-specific tolerance thresholds.

In addition to reporting ^225^Ac-specific absorbed-dose metrics, general dosimetry reporting standards should be applied [82]. Individual patient data should be presented, and when doses are expressed in Gy/MBq, the administered activity must be stated. If dose–volume histograms or isodose curves are produced, their results should be included to characterize dose heterogeneity. All measurement uncertainties, as well as those propagated through subsequent calculations, must be quantified. The corresponding physical absorbed-dose values should accompany radiobiological modelling outputs.

In most studies, toxicity guidance has relied on maximum tolerated dose (MTD) thresholds. MTD thresholds used in radionuclide therapy are largely extrapolated from external beam radiotherapy (EBRT) or from other radionuclide therapies, and may not be directly applicable to ^225^Ac-based TAT [83]. These thresholds originate from very different radiation types, dose rates, temporal delivery profiles, and radiobiological mechanisms. As a result, simply adopting EBRT-derived organ tolerance limits overlooks the high LET and elevated RBE of α-emitters, as well as patient-specific susceptibility factors.

Doses in ^225^Ac radiopharmaceutical therapy are often compared with MTD thresholds derived from EBRT or other radiopharmaceutical therapies. Although MTD can provide guidance, these thresholds may not be directly applicable due to the high-LET emissions, subcellular dose deposition, and unique pharmacokinetics of ^225^Ac. The Food and Drug Administration (FDA) has highlighted that relying solely on MTD or historical organ dose limits may be insufficient for radiopharmaceutical therapies (RPTs), emphasizing that trial dosing should account for cumulative, delayed, and potentially irreversible toxicities [84]. Consideration of organs at risk, including the kidneys, salivary glands, and red marrow, remains critical in ^225^Ac therapy, as these tissues may limit the number of safely deliverable treatment cycles. The lack of α-emitter-specific clinical MTD adds further uncertainty, highlighting the need for careful interpretation of absorbed doses and potential cumulative toxicity in these critical organs. Patient-specific dosimetry, incorporating organ- and tumour-level absorbed doses, radiobiological weighting, and individualized risk factors, is recommended to optimize safety and efficacy. While MTD data can provide context, FDA guidance stresses using a totality-of-evidence approach for dosing decisions, and validation of these dosimetry-driven strategies for ^225^Ac is ongoing.

### 1.7. Actinium-225 Clinical Imaging Protocols for Dosimetry Based on Decay Emission

Quantitative imaging of ^225^Ac and its progeny (Figure 2) remains a central challenge in TAT due to the low administered activities and limited photon yield of α-emitting radionuclides [67,70,71,72,79,85]. Key gamma emissions for SPECT imaging include 440 keV (25.9%), 218 keV (11.4%), and 78–92 keV (characteristic X-rays), with HEGP employed to optimize spatial resolution and minimize septal penetration [67,70,71,72,79,85]. Multi-window acquisition strategies, typically including the main photopeaks and optional adjacent scatter windows, are required to correct for scatter, crosstalk, and other image-degrading effects, enabling accurate activity quantification [67,70,71].

Clinical and preclinical studies have explored various acquisition protocols. Liubchenko et al. [70] performed SPECT/CT at 24- and 48-hours post-injection of ^225^Ac-PSMA-I&T, using 16 projections per head with 210 s per projection, capturing the 440 keV and 218 keV peaks with window widths of 20–50%. Multi-isotope studies combining ^177^Lu/^225^Ac SPECT/CT demonstrated that imaging the 440 keV peak of ^213^Bi alongside 208 keV for ^177^Lu is feasible, with 16 projections per head and ~3.5 min per projection, providing adequate image statistics for organ- and lesion-level dosimetry [79]. Phantom studies using Jaszczak, National Electrical Manufacturers Association (NEMA) IEC, and 3D-printed tumour models confirmed that quantitative SPECT/CT can achieve activity quantification within 10% uncertainty, with iterative reconstruction algorithms including attenuation, scatter, and point spread function (PSF) modelling being essential [67,85].

Imaging at multiple time points is critical for accurate dosimetry, reflecting the pharmacokinetics of both parent ^225^Ac and its daughters ^213^Bi and ^221^Fr. Effective half-lives in kidneys and lesions range approximately 24–38 hours, and imaging at 24 and 48 hours post-injection captures key dynamics for TIAC calculations [70]. Urine analysis and imaging studies indicate modest redistribution of ^213^Bi from ^225^Ac via α-recoil, with only minor impact on absorbed doses (<10%) [70,72]. Bauer et al. [63] demonstrated that the ^134^Ce/^134^La positron emission tomography (PET) pair enables assessment of ^225^Ac progeny redistribution, showing that internalizing tracers retain daughter isotopes at the target site and provide a method to monitor and potentially mitigate α-decay recoil in vivo.

Recent studies, such as the ACTION-1 trial with RYZ101 (^225^Ac-DOTATATE), extend these principles to somatostatin receptor 2-expressing neuroendocrine tumours [71]. SPECT/CT acquisitions were performed at 4 ± 1 h, 24 ± 2 h, and 168 ± 24 h post-infusion for both Cycle 1 and Cycle 4, using HE collimators and three energy windows (92, 218, and 440 keV; widths 25%, 20%, 20%, respectively). Dual-radionuclide reconstruction allowed simultaneous imaging of ^221^Fr and ^213^Bi, enabling precise quantification of organ (liver, kidneys, spleen, red bone marrow) and tumour uptake. The mean RBE-weighted absorbed dose coefficients for Cycle 1 in four source organs were 1.1, 0.45, 0.30, and 0.032 Gy/MBq for spleen, kidneys, liver, and red marrow, respectively, while selected tumours exhibited dose coefficients of 1.0–4.8 Gy/MBq. Notably, ^213^Bi largely remained associated with the DOTATATE delivery agent, with only minor redistribution to kidneys, confirming that imaging both daughters provides a reliable surrogate for ^225^Ac biodistribution [71].

Collectively, published work demonstrates the feasibility of quantitative ^225^Ac SPECT/CT for clinical dosimetry across a range of acquisition protocols (Table 2). Energy windows centred at 440 keV, 218 keV, 78 keV or 92 keV have been used, typically with a HE collimator, 15–60 projections per head, and projection times ranging from 30 s to 210 s depending on angular sampling. Multiple time-point imaging, commonly at early (~4 h), intermediate (~24–48 h), and late (168 h) post-injection, enable the determination of TIACs.

**Figure 7:**
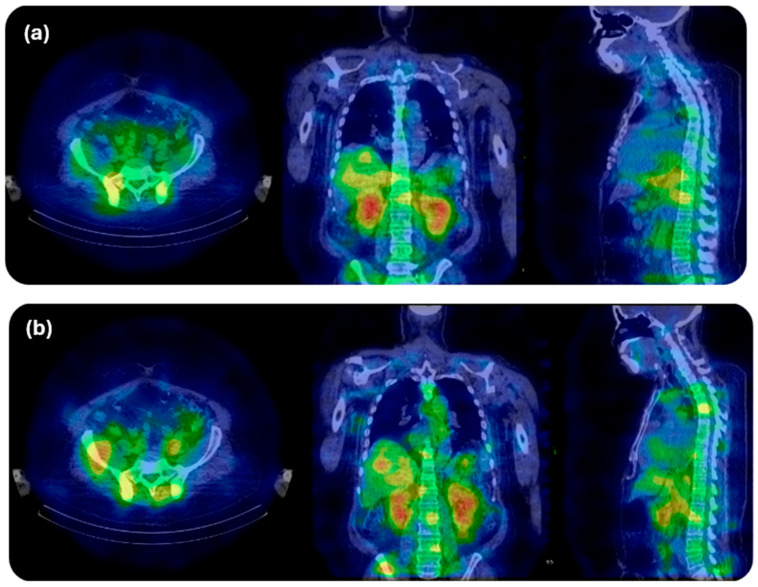
SPECT/CT feasibility for clinical image activity quantification of ^225^Ac-derived emissions. (**a**) Acquisition using the 218 keV photon emission, arising primarily from the decay of ^217^Fr. (**b**) Acquisition using the 440 keV photon emission, originating predominantly from the decay of ^213^Bi.

Figure 7 demonstrates the feasibility of clinical ^225^Ac SPECT/CT for activity quantification in dosimetry at 24 h post-injection following administration of 7.5 MBq. Acquisitions employed 20% energy windows centred at 218 keV and 440 keV, with 60 projections per head and 30 s per projection, reconstructed with attenuation, scatter, and resolution-recovery corrections. These results confirm that direct quantitative imaging of ^225^Ac for dosimetry is achievable in a clinical setting and highlight the potential for harmonized acquisition and reconstruction protocols to standardize image activity quantification for dosimetry.

Clinical implementation of image-based dosimetry for ^225^Ac remains limited by low administered activities, restricted photon statistics, and complex decay schemes, which affect quantitative robustness. While SPECT using ^213^Bi and ^221^Fr emissions enables patient-specific dosimetry, protocol optimization and validation remain essential, and surrogate PET approaches provide mechanistic insight but do not directly yield absorbed α-dose. These limitations directly influence treatment planning, including uncertainty in organ-at-risk dose constraints and the number of safely deliverable therapy cycles.

When combined with RBE-weighted absorbed dose calculations, these imaging strategies support precise organ- and lesion-level dosimetry, improving the safety and efficacy evaluation of ^225^Ac targeted therapies in metastatic prostate cancer and SSTR2+ neuroendocrine tumours [67,70,71,72,79,85].

### 1.8. General Principles of Absorbed Dose Computation for Actinium-225 Dosimetry

Accurate dosimetry for ^225^Ac requires methods that explicitly account for its multistep decay chain, short-range α-particles, daughter redistribution, and non-negligible branching ratios. α-emitting radionuclides deposit energy over microscopic ranges (40–100 μm) [7]. Therefore, conventional organ-level and S-value-based approaches must be applied with special considerations.

#### 1.8.1. Core Dosimetry Assumptions for α-Emitters

For organ- and tumour-level dose estimation, activity within a defined source region is generally assumed to be uniformly distributed, and cross-organ dose contributions from α-particles and electrons are considered negligible because of their extremely short range in tissue [86,87,88,89]. In contrast, γ-emissions can contribute cross-organ dose and must be treated separately. However, α-emitters such as ^225^Ac require important modifications to this conventional framework because they generate multiple daughter radionuclides that emit additional α-, β-, and γ-radiations. As a result, a substantial portion of the absorbed dose arises not only from the parent radionuclide but also from successive daughter decays, particularly when short-lived daughters decay locally at the site of formation.

#### 1.8.2. Limitations of Standard S-Value Methods for ^225^Ac

For α-emitting radionuclides, S-values calculated solely for the parent nuclide can be incomplete and may substantially underestimate the true absorbed dose. For example, an S-value assigned to ^213^Bi excludes the dominant α-emission from its daughter ^213^Po, even though ^213^Po contributes roughly 98% of the total α-particle energy and has an extremely short half-life of only 4 ms [90]. In addition, branched decay pathways require the dosimetry to incorporate the relative yields and mean energies of all emissions produced throughout the decay chain. Consequently, accurate α-dose estimation relies on absorbed-fraction-based calculations that integrate contributions from each relevant daughter, rather than on parent-only S-values.

#### 1.8.3. Formal Absorbed-Dose Framework for α-Emitters

Using the updated MIRD schema [91], the absorbed dose to a target region rT from emission type x (α−particles, electrons or photons) is as follows:

Dx(rT, TD)=Ã(rS, TD) · Σi Di^x φ(rT ← rS; Ei^x)/M(rT)

where Ã(rS, TD) = TIA in the source region, Di^x = mean energy emitted per transition for the i-th emission, φ = absorbed fraction for that energy and emission type, and M(rT) = mass of the target region and x ∈ {α, e, γ}.

For dosimetry, the absorbed fraction for α-particles and electrons is generally assumed to be approximately 1, whereas photons require the use of tabulated photon absorbed-fraction values.

#### 1.8.4. Accounting for Daughter Emissions in ^225^Ac Dosimetry

For accurate α-dosimetry of ^225^Ac, it is essential to account for the entire decay chain, as the radionuclide produces multiple α- and β-emissions through its sequence of daughters. Each radionuclide in the chain must be included, with careful application of branching ratios and energy yields to correctly quantify the total energy deposited. Short-lived daughters, such as ^213^Po (T_1/2_ = 4 ms), decay essentially at the site of formation, contributing localized α-dose, whereas longer-lived daughters, including ^213^Bi (T_1/2_ = 45.6 min) [58], may redistribute within the body, necessitating region-specific assignment of TIACs to accurately reflect their contribution to the absorbed dose.

#### 1.8.5. Time-Integrated Activity Coefficients

TIACs constitute the foundation for absorbed dose calculations. They are obtained by numerically integrating pharmacokinetic models that describe the temporal distribution of radioactivity in each source region. For α-emitting radionuclides such as ^225^Ac, the TIACs of daughter radionuclides are allocated according to their site of decay or, when redistribution occurs, to the corresponding tissue compartments [92]. Each daughter TIAC is further adjusted by its net decay yield to accurately represent the contribution of all sequential decays to the total absorbed dose.

#### 1.8.6. Practical Dose Calculation for ^225^Ac in Clinical Dosimetry

Modern absorbed dose calculations integrate several key components to achieve accurate dosimetry for α-emitting radionuclides such as ^225^Ac. These calculations combine TIACs for each parent and daughter radionuclide with emission-specific absorbed fractions, for which ICRP voxel phantoms are recommended [93], and sum the contributions from α, β, and γ emissions to determine the total absorbed dose. Computational tools such as 3D-RD-S (Radiopharmaceutical Imaging and Dosimetry, LLC, Rapid, Baltimore, MD) facilitate absorbed dose calculations by accounting for daughter redistribution and organ-specific α-particle absorbed fractions [94]. These tools also incorporate updated ICRP voxel phantom data, enabling precise, organ-level dosimetry for radionuclides with complex decay chains.

#### 1.8.7. Relative Biological Effectiveness Considerations and Biological Effective Dose

RBE considerations are particularly important for ^225^Ac TAT due to the high LET of α-particles. RBE is defined as the ratio of the absorbed dose of a reference radiation (e.g., X-rays, γ-rays, β-particles), Dr(x), required to elicit a specific biological effect, x, to the absorbed dose of the test radiation, Dt(x), needed to produce the same effect, as follows:


RBE(x)=Dr(x)/Dt(x)

Although α-particles generally exhibit an RBE of approximately five [95,96], some dosimetry frameworks report absorbed dose without applying RBE correction, allowing user-specific weighting of α-contributions. Experimental studies highlight substantial variability in RBE depending on biological endpoint and cell geometry [97]. Rumiantcev et al. [98] reported RBE values at zero dose for ^225^Ac ranging from 9.33 to 10.84 across cells with different geometries, using event-by-event Monte Carlo (MC) simulations and the yield of radiation-induced double-strand breaks as the biological endpoint. In contrast, our study focused on cell survival fraction and calculated RBE individually for each radionuclide in the ^225^Ac decay chain, accounting for potential redistribution of decay products, which may explain differences from their reported values. Similarly, Ruigrok et al. [99] measured an RBE of 4.2 for ^225^Ac-PSMA-I&T relative to ^177^Lu-PSMA-I&T using clonogenic survival assays. Using the empirical formula from MIRD Pamphlet No. 21 [91], the RBE for ^225^Ac was calculated as approximately 6.1, illustrating differences across experimental approaches and biological systems.

These findings underscore the importance of incorporating cell- and tissue-specific radiosensitivity into dosimetry for ^225^Ac TAT. Once the absorbed dose for a tumour or organ has been determined using nuclear medicine imaging or pharmacokinetic models, the total number of decays for each radionuclide within the region of interest can be calculated. By applying the weighting method proposed by Zaider et al. [100], the radiobiological parameters and RBE of the mixed radiation field can be determined, enabling computation of the RBE-weighted dose $D_{RBE}$ as follows:

$$D_{RBE}\left(rT,TD\right)=RBE\alpha \cdot D\alpha \left(rT,TD\right)+RBEe\cdot De\left(rT,TD\right)+RBE\gamma \cdot D\gamma \left(rT,TD\right)$$

where $D\alpha \left(rT,TD\right),De(rT,TD)$ and $D\gamma \left(rT,TD\right)$ are the absorbed doses from α-particles, electrons, and photons, respectively, calculated using TIACs and absorbed fractions. RBEα, RBEe, and RBEγ are the relative biological effectiveness factors for α-particles, electrons, and photons, respectively (commonly, RBEe=RBEγ=1).

This approach allows precise assessment of therapeutic efficacy and normal tissue toxicity by integrating the contributions from α-, β-, and photon emissions in a biologically relevant manner, tailored to the radiosensitivity of the tissue or cell type under consideration [97].

### 1.9. Biologically Effective Dose in Actinium-225 Targeted Alpha Therapy

Biologically effective dose (BED) is a radiobiological metric that quantifies the biological effect of a delivered radiation dose, integrating not only the total physical dose but also the dose rate, fractionation schedule, and tissue-specific radiosensitivity. It provides a standardized framework for comparing different radiation modalities or treatment regimens, including external beam radiotherapy (EBRT) and radionuclide therapies.

For fractionated or single-dose treatments, BED is typically derived from the linear-quadratic (LQ) model:

BED=D ∗ (1+d/(α/β))

where D is the total physical dose, d is the dose per fraction, and α/β represents the radiosensitivity parameter of the tissue or cell type.

For continuous, low-dose-rate or exponentially decreasing dose rates characteristic of TRT, the BED formalism can be extended to account for ongoing radiation delivery and biological repair kinetics.

In high LET radiation, such as alpha particles from ^225^Ac, the RBE must be considered, reflecting the enhanced biological damage per unit absorbed dose when compared with low-LET radiation:


BED_α=RBE ∗ BED_physical

This approach allows absorbed dose measurements from beta-emitters (e.g., ^177^Lu) to be converted to the equivalent biological effect for alpha-emitting radionuclides, including contributions from all daughter isotopes in the decay chain [101]. BED calculations for TAT can then be integrated with normal tissue complication probability models derived from EBRT to evaluate potential toxicity and guide treatment optimization.

For alpha emitters, an RBE of 5 is often assumed in BED calculations [71]. However, when using the D_0_ values determined for X-rays in the same cell line as the alpha-emitter studies, a linear correlation between RBE and the initial alpha-particle energy was observed, with initial energies ranging from 5 to 8.5 MeV [90]. This indicates that BED calculations for alpha-emitting radiopharmaceuticals may be refined by accounting for the specific alpha-particle energy, rather than assuming a fixed RBE, thereby improving the accuracy of radiobiological dose estimation.

### 1.10. Surrogate Imaging for Actinium-225 Radiopharmaceutical Therapy Dosimetry

Recent technological advances have enabled low-count quantitative SPECT/CT imaging using the ^213^Bi 440 keV (25.9%) and ^221^Fr 218keV gamma emissions. This development permits patient-specific dosimetry in clinical settings and is currently undergoing clinical validation [79,102]. Dosimetry for [^225^Ac]Ac-PSMA RLT is inherently more complex than for [^177^Lu]Lu-PSMA, largely because ^225^Ac and its daughters emit few suitable gamma photons for routine quantitative imaging. The short path length and high LET of alpha particles further complicate spatial dose estimation, particularly at the subcellular level.

Given these limitations, surrogate approaches have traditionally been employed, including pre- and post-treatment PET imaging with [Gallium-68]Ga-PSMA, combined with organ function monitoring and haematological assessments [67,79]. These surrogates provide indirect estimates of biodistribution and absorbed dose but do not capture the full spatial heterogeneity inherent to alpha emitters. From a radiobiological perspective, alpha particles exhibit markedly higher RBE than beta emitters, often assumed to be around five times that of ^177^Lu [103]. As a result, tumour effects are achieved at substantially lower administered activities, approximately 100 kBq of [^225^Ac]Ac-PSMA is estimated to produce a comparable therapeutic effect to 7.4 GBq of [^177^Lu]Lu-PSMA [104]. Dosimetry modelling at the subcellular scale indicates that ^225^Ac can deliver effective doses that are several thousand-fold higher than those from β-emitters in micrometastatic disease or isolated cells, underscoring its exceptional capacity to eradicate minimal residual disease [104].

### 1.11. Harmonisation of Activity Quantification for ^225^Ac Dosimetry—Provisional and Subject to Ongoing Validation

Accurate and harmonised activity quantification is a prerequisite for reliable patient-specific dosimetry in TAT with ^225^Ac and its derivatives. Absolute quantification enables meaningful dose–response analyses, improved prediction of toxicity, and rigorous treatment follow-up, but achieving consistent results across centres requires standardised procedures for acquisition, calibration, and reconstruction [105,106,107,108]. For SPECT, the challenge is amplified relative to PET because of the need for a collimator, the detector trajectory, the need for a mechanical, angle-dependent detector motion around the patient (i.e., the SPECT acquisition orbit), and more complex scatter and attenuation settings; consequently, quantification depends critically on system- and protocol-specific factors, including calibration traceability and reconstruction protocols that include activity quantitative corrections [73,107,109]. Inter-system variability has been repeatedly documented, for objects substantially larger than the spatial resolution, absolute quantification can be achieved within ~10% on some systems, whereas smaller structures show substantially larger errors that mandate partial-volume correction and harmonised reconstruction [110,111,112]. These observations motivate vendor-neutral standards and accreditation efforts (e.g., EARL-style initiatives and the emerging ^177^Lu accreditation work) to permit reliable multi-centre dosimetry and quantitative studies [73,113,114].

For ^225^Ac specifically, harmonisation must address the low administered activities and limited photon yield of alpha emitters [67,70,71,72,79,85]. Practical protocol essentials that should be standardised include collimator selection (HEGP or HE), energy window selection (primary photopeaks at ~440 keV and ~218 keV with adjacent scatter windows and, where appropriate, auxiliary low-energy windows), window widths, scan time per projection and number of projections, and the timing of serial imaging to record relevant pharmacokinetics (e.g., early ~4 h, 24 h, and late 48–168 h) for robust TIAC estimation [67,70,71,72,79,85]. Recovery (explicitly applied) and volumetry methods used for VOI delineation must be documented and harmonised because segmentation choices substantially affect activity estimates and propagated absorbed dose uncertainties [78,115]. Calibration procedures must be traceable to metrological standards to reduce inter-centre bias and enable aggregation of dosimetry data accuracy [73,116].

Reconstruction protocols are a central element of harmonisation. Iterative reconstruction must routinely incorporate CT-based attenuation correction, accurate scatter correction (for example triple-energy-window or equivalent methods adapted for the complex ^225^Ac spectrum), and explicit modelling of the CDR, including septal penetration and high-energy interactions relevant to the 440 and 218 keV emissions, such as those applied in quantitative single-photon emission computed tomography [73,108,117]. Resolution recovery (point-spread-function modelling) and noise-regularisation strategies should be specified and validated, as matrix size, voxel sampling, and post-filtering materially affect quantitative accuracy and inter-scanner comparability [111,112,118,119]. Where feasible, reconstruction should be validated using physical phantoms (Jaszczak, NEMA IEC and task-specific 3D-printed phantoms) to derive RCs for PVC and to demonstrate quantification within stated uncertainty bounds [67,85]. Multi-window acquisition strategies and dual-radionuclide reconstruction approaches that explicitly account for crosstalk between peaks (e.g., combined ^213^Bi/^221^Fr and ^177^Lu in mixed-isotope studies) should be standardised and their limitations documented [79].

Time-series imaging protocols and pharmacokinetic modelling must also be harmonised. Imaging at multiple post-injection time points that reflect the effective biological half-times of organs and lesions (commonly ≈ 24–38 h for kidneys and lesions) is required to produce robust TACs and TIACs for dosimetry, mono-exponential fits are commonly used when data are sparse, whereas multi-exponential fitting should be reserved for cases with at least four well-spaced time points [70,120,121,122]. Uncertainty reporting should be standardised: centres should report individual patient results (not only cohort means), the administered activity when expressing Gy/MBq, and the propagated measurement and modelling uncertainties for TIACs and absorbed doses in accordance with MIRD Pamphlet No. 23 and related guidance [73]. When radiobiological metrics (e.g., RBE-weighted BED) are reported, the underlying physical absorbed-dose quantities must also be provided, and the number of significant digits should be consistent with the estimated uncertainties.

Finally, harmonisation requires coordinated multi-centre validation. Early clinical and phantom studies demonstrate that quantitative ^225^Ac SPECT/CT is feasible with HE collimator, multi-window acquisition, iterative reconstruction with attenuation, scatter and PSF/CDR modelling, and appropriate scan durations and time points, these procedures can deliver TIACs suitable for RBE-weighted dose estimation [67,70,71,72,79,85]. Biological and microdosimetric uncertainties arise from the common assumption of a fixed RBE (=5) for TAT. However, this simplified approach does not fully reflect the complex radiobiological properties of alpha radiation. The biological effect is influenced by several factors, including dose rate, radiation track structure, subcellular activity distribution, and tissue-specific radiosensitivity, which may vary substantially across organs and tumour microenvironments. As a result, higher biological effectiveness than commonly assumed may be plausible in certain contexts [6,79]. Incorporating RBE-weighted dose estimates that account for these parameters could therefore enhance the biological relevance and interpretation of absorbed dose calculations, providing a more mechanistically informed framework for dosimetry in targeted alpha therapy. ^225^Ac systematic validation across scanner platforms, reconstruction implementations, and clinical sites is essential and is underway.

### 1.12. Microdosimetry in Actinium-225 Targeted Alpha Therapy

Accurate dosimetry of α-emitting radionuclides, particularly ^225^Ac, requires microdosimetric considerations due to the highly localized and stochastic energy deposition of α-particles. Unlike low-LET radiation, where numerous independent events are needed to elicit a measurable biological effect, a single α-particle traversing a cell nucleus can deliver a lethal dose, rendering the mean absorbed dose potentially misleading [123,124]. Classical MIRD-based methods provide average dose estimates for compartments such as the nucleus, cytoplasm, or cell surface using tabulated S-values. Nevertheless, these approaches do not capture the stochastic variations intrinsic to high-LET α-particle irradiation. Microdosimetry explicitly accounts for specific energy (energy per unit mass) and lineal energy (energy per unit path length), the number of particle traversals, and their geometric relationships to the target [125,126,127]. Microdosimetric evaluation becomes critical when stochastic variations exceed 20% of the mean local dose, as can occur in small nuclei or low-dose regions [128]

Several studies have demonstrated the necessity of microdosimetry in α-radiopharmaceutical therapy. Hofmann et al. [129] emphasized its utility in evaluating heterogeneous nuclear energy deposition and predicting biological effects. Li et al. [130] highlighted the importance of subcellular dosimetry in TAT. The MIRD committee developed MIRDcell [130,131] an analytical platform validated against MC simulations, Electron Gamma Shower, version 4 for calculating cellular doses and biological outcomes [132]. Lee et al. [133] quantified the impact of radionuclide internalization on nuclear dose, while Koniar et al. [134] modelled intracellular dose distributions of ^225^Ac and its progeny across variable subcellular distributions. Sato et al. [17] extended PHITS to compute microdosimetric doses in TAT and incorporated the microdosimetric kinetic model to estimate dose-dependent RBE and EQD_X(α/β).

Together, these approaches integrate time-dependent activity, emission-specific absorbed fractions, and stochastic energy deposition, providing a biologically relevant framework for assessing both therapeutic efficacy and normal tissue toxicity. Microdosimetry complements RBE-weighted absorbed dose calculations by resolving heterogeneity at the cellular and subcellular scale, which is particularly critical for small, sparsely irradiated targets where conventional mean-dose estimates may underestimate biological effects. This integrated methodology is therefore indispensable for accurate, clinically relevant dosimetry in ^225^Ac targeted alpha therapy.

### 1.13. Future Directions in Tiered Dosimetry and Technological Advancements

Accurate, patient-specific dosimetry for ^225^Ac RPT remains a complex yet critical component for optimizing therapeutic efficacy while minimizing toxicity. Clinical implementation benefits from a tiered approach that balances dosimetry precision with patient convenience. Recent studies support the use of reduced post-therapy imaging schedules, including single- and dual-time-point acquisitions, which achieve acceptable TIA errors while limiting patient burden [135,136,137,138]. Mixed-model approaches using historical cohorts further reduce bias and outliers compared with conventional single-time-point methods, enhancing accuracy while lowering imaging burden [136]. Advanced reconstruction techniques, such as 3-D OSEM, have demonstrated improved spatial resolution, quantitative accuracy, and noise suppression for ^225^Ac SPECT/CT imaging, particularly when combined with corrections for attenuation, scatter, CDR, and noise regulation [139].

AI-based methods have been shown to improve multiple steps in the dosimetry workflow for radiopharmaceutical therapy. Convolutional neural networks (CNNs) and deep learning (DL) models can denoise raw projection data, reducing noise-induced variability and improving quantitative SPECT image quality [140,141]. These models also enhance multimodal and multi-time-point image registration, leading to more accurate alignment of anatomical and functional data [141]. AI-driven segmentation tools have been demonstrated to reduce intra- and inter-observer variability in delineating organs and lesions, shortening analysis time and standardizing volume definition [142,143,144,145]. Finally, AI facilitates voxel-level time-activity curve fitting, enabling precise estimation of TIA for individual organs and tumours while minimizing manual intervention [146,147,148]. Collectively, these applications support more reliable, patient-specific dosimetry in clinical settings.

Clinical dosimetry should focus on optimizing imaging acquisition times to accurately reflect radionuclide pharmacokinetics, particularly for organs with long effective half-lives, where late-time-point imaging is essential [122,135,149,150,151]. Recent advancements in gamma camera technology, particularly cadmium–zinc–telluride (CZT), have transformed patient-centred dosimetry. CZT-ring-based detector systems, exemplified by the StarGuide SPECT/CT (GE Healthcare) and Veriton SPECT/CT (Spectrum Dynamics, Israel), provide markedly improved performance compared with conventional Anger-type cameras. These include superior energy resolution (5–6% FWHM versus 8–10% FWHM), approximately threefold higher system volume sensitivity (SVS) (~520 cps/MBq), and enhanced spatial resolution (~4 mm FWHM) [152]. These intrinsic properties allow for more accurate activity quantification, particularly when combined with CFs or SVS, enabling SUV-based measurements and more robust dosimetry calculations. Advanced reconstruction algorithms incorporating noise regularization further enhance image quality and quantitative reliability [153]. Additionally, the improved sensitivity and detector geometry enable rapid whole-body SPECT imaging, achieving complete scans in 12–15 min without compromising quantitative accuracy. Such capabilities are particularly important for theranostic applications, where robust dosimetry is critical for treatment response assessment and individualized therapy planning. Challenges remain, including septal penetration, high-energy photon handling, and detector dead-time effects at elevated count rates, which must be addressed to ensure reliable quantitative performance across clinical scenarios. By combining enhanced quantitative capabilities with rapid imaging and robust reconstruction methods, these systems provide a platform for advancing theranostic applications and improving patient-centred nuclear medicine. Continued refinement and standardization will be essential to fully realize their potential in precision dosimetry and personalized treatment strategies.

Future studies leveraging the ^134^Ce/^134^La PET in vivo generator hold significant potential to advance quantitative theranostics with α-emitters. This radionuclide pairing enables noninvasive in vivo investigation of α-decay recoil, tracer internalization kinetics, and receptor trafficking, and is particularly well suited to internalizing targeting vectors. Previous work has demonstrated that cerium-134/lanthanum-134 (^134^Ce/^134^La) PET can be used to assess the redistribution of ^225^Ac progeny, showing that internalizing tracers are more effective at retaining daughter isotopes at the target site and thereby offering a strategy to monitor and potentially mitigate recoil-related effects [63]. Taken together these capabilities position ^134^Ce as a promising theranostic surrogate for ^225^Ac, offering a platform for the mechanistic studies of tumour microenvironment dynamics, tracer behaviour, and personalized dose optimization in future clinical translation.

## 2. Conclusions

In conclusion, patient-specific ^225^Ac dosimetry requires a comprehensive, multidisciplinary workflow integrating harmonized quantitative imaging, advanced reconstruction, computational analysis, and mechanistic theranostic evaluation. Standardized SPECT acquisition and decay-chain-resolved absorbed-dose calculations, combined with RBE-weighted evaluation, provide a robust framework for biologically informed, individualized dose assessment. AI-driven methods enhance precision and efficiency across the dosimetry workflow. Complementing these advances, CZT-based gamma cameras enable high-accuracy quantitative imaging and rapid whole-body acquisition, supporting precise activity measurement and patient-centred dosimetry. Additionally, the ^134^Ce/^134^La PET in vivo generator provides a unique theranostic platform to noninvasively monitor ^225^Ac progeny redistribution, evaluate alpha-decay recoil, and investigate tracer internalization and receptor trafficking, particularly for internalizing vectors. Collectively, these innovations support mechanistically informed, patient-centred TAT, enabling optimized treatment planning and precise response assessment. Continued standardization and validation of imaging, reconstruction, and dosimetry workflows will be critical to fully realize the potential of precision nuclear medicine and to translate these advancements into reproducible, individualized clinical care.

AI-based methods have demonstrated clear potential to improve quantitative ^225^Ac dosimetry through projection denoising, image registration, automated segmentation, and voxel-level TACs, but wider clinical adoption is currently limited by the need for standardized training datasets, regulatory validation, and seamless integration into clinical workflows. CZT-based gamma cameras offer improved sensitivity and energy resolution for low-count α-emitter imaging; however, their routine clinical adoption remains constrained by system availability, cost, and technical challenges such as septal penetration, high-energy photon handling, and detector dead-time effects. Continued technical optimization and multicentre validation are required before these technologies can be broadly implemented in clinical dosimetry practice.

## Figures and Tables

**Table 1:** Comparative overview of ^225^Ac production pathways: yield, purity, radiological characteristics, and clinical considerations.

Production Route	Yield/Availability	Isotopic Purity	Key Radiological and Dosimetry Characteristics	Clinical Theranostic Considerations	Advantages	Limitations/Challenges	Current Status
^229^Th radionuclide generator	Moderate; clinically validated	Very high purity. Essentially no ^227^Ac	^225^Ac T_½_ = 9.92 d; α-emitter (Eα ≈ 5.8 MeV); decays to ^221^Fr → ^217^At → ^213^Bi (T_½_ = 45.6 min, α-emitter); high specific activity (~0.2–0.3 TBq/µmol)	Provides high-quality ^225^Ac for targeted α-therapy; ^213^Bi available for theranostic applications; well-defined dosimetry due to negligible long-lived impurities	Clinically established generator system; carrier-free ^225^Ac; dual-use for α-therapy and theranostics	Limited ^229^Th global supply; long parent half-life limits production scale	Active: JRC Karlsruhe (DE), ORNL (USA), IPPE (RU); supply constrained
^232^Th spallation	High; multi-Curie scale	Moderate purity. ^227^Ac impurity ~0.1–0.2%	^225^Ac T_½_ = 9.92 d; α-emitter; ^227^Ac T_½_ = 21.77 y contributes long-term dose; α-energy ~5–6 MeV; specific activity slightly reduced due to ^227^Ac	Suitable for high-volume clinical supply; dosimetry affected by long-lived ^227^Ac; careful radiation safety and waste management required	Capable of producing multi-Curie quantities; meets future demand	Trade-off between yield and isotopic purity; regulatory and waste management challenges due to ^227^Ac	Implemented at U.S. Tri-Labs (ORNL, LANL, BNL); large-scale production
^226^Raproton irradiation	Medium–high; ~5 GBq per 50 mg ^226^Ra, 24 h at 100 μA	High purity. Negligible ^227^Ac	^225^Ac T_½_ = 9.92 d; α-emitter; short-lived impurities ^226^Ac T_½_ = 29 h, ^224^Ac T_½_ = 2.9 h decay during post-irradiation cooling; high specific activity	Produces clinically suitable ^225^Ac for α-therapy; predictable dosimetry; minimal long-lived impurity contribution	High-purity ^225^Ac; scalable; cyclotron-based	Requires handling of ^226^Ra and gaseous ^222^Rn; specialized cyclotron and radiochemistry infrastructure	Widely used research and production route; mature
^226^RaGamma irradiation	Low; not demonstrated at clinical scale	Potentially high purity. Unproven at scale.	^225^Ac T_½_ = 9.92 d; α-emitter; intermediate ^225^Ra T_½_ = 14.9 d contributes to ingrowth dose	Theoretically capable of producing high-purity ^225^Ac; dosimetry is predictable once the process is optimized	Non-proton alternative; potential for high-purity production	Limited by gamma source intensity; no proof-of-principle for high-activity clinical-scale production	Exploratory/proof-of-concept stage
References	[27,28,29,49,54,55]						

**Table 2:** Summary of clinical and phantom ^225^Ac SPECT imaging protocols for dosimetry.

Study/Reference	Photopeak energy (keV) and Window Width (%)			Imaging Time Points Post-Injection	Model and Manufacturer	Reconstruction Algorithm	Reported Absorbed Doses/Key Observations
	Bismuth-213	Francium-221	X-Ray Emissions				
Liubchenko et al. [70]	440 keV (20%)	218 keV (20%)	78 keV (50%)	24 h, 48 h	Siemens Symbia T2 SPECT/CT (Siemens Medical Solutions, Erlangen, Germany)	In-house MAP-MLEM algorithm	Mean kidney and lesion absorbed doses. ^221^Fr & ^213^Bi images: 0.17 ± 0.06 Sv_(RBE=5)_/MBq & 0.36 ± 0.1 Sv_(RBE=5)_/MBq Either ^221^Fr/^213^Bi images: 0.16 ± 0.05/0.18 ± 0.06 Sv_(RBE=5)_/MBq & 0.36 ± 0.1/0.38 ± 0.1 Sv_(RBE=5)_/MBq
Delker et al. [79]	440 keV (10%)	Not imaged	Not imaged	24 h	Symbia Intevo T16 SPECT/CT (Siemens Healthineers, Erlangen, Germany)	In-house MAP-EM algorithms	Kidney and lesion absorbed doses ^225^Ac: 0.28 ± 0.14 & 0.22 ± 0.21 Sv_(RBE=5)_MBq
Gosewisch et al. [72]	440 (20%)	218 keV (10%)	Not imaged	24 h	Siemens Symbia Intevo T16 SPECT/CT (Siemens Healthineers, Erlangen, Germany)	MAP algorithm	Left kidney, right kidney and lesion absorbed doses ^177^Lu: 0.27, 0.24 & 0.38 Gy/GBq 0.18, 0.1 & 0.26 Sv_(RBE=5)_/MBq
Tulik et al. [67]	444 keV (10%)	217 KeV (10%)	78 keV (20%)	Phantom study	Siemens Symbia T6 SPECT/CT (Siemens Healthineers, Germany)	OSEM FLASH 3D algorithm (Siemens Healthineers)	Phantom calibration (Jaszczak and 3D-printed tumour model), activity quantification within 10% accuracy
Benabdallah et al. [85]	410 keV (±6.1%) [GE Healthcare] 444 keV (±5%) [Siemens Healthineers]	218 keV (±8%) [GE Healthcare] 217 keV (±8%) [Siemens Healthineers]	80 keV (±20%) [GE Healthcare] 82 keV (±20%) [Siemens Healthineers]	Phantom study	Discovery 670 SPECT/CT (GE Healthcare) and Siemens Symbia T6 SPECT/CT (Siemens Healthineers, Germany)	2D-OSEM algorithm (GE Healthcare) 3D-OSEM algorithm (Siemens Healthineers)	Evaluated detection limits, reconstruction, and sensitivity across multiple gamma cameras
Sgorous et al. [71]	440 keV (20%)	218 keV (20%)	92 keV (25%)	4 ± 1 h, 24 ± 2 h, 168 ± 24 h	Manufacturer unspecified	Dual-radionuclide quantitative SPECT reconstruction used	Weighted absorbed dose coefficients for source organs and lesions (Mean RBE = 5): Spleen (1.1 Gy/MBq), kidneys (0.45 Gy/MBq), liver (0.30 Gy/MBq), red marrow (0.032 Gy/MBq), 11 Tumours (1.0–4.8 Gy)
Polson et al. [69]	440 keV (20%)	218 keV (20%)	Not imaged	6 h, 20.5 h, 76.5 h, 284.6 h	Simulation study (SIMIND MC program)	MLEM algorithm	Time-integrated activity and uncertainty for 3 lesions: Lesion 1 (11.2 MBq·h ± 14.02%), Lesion 2 (0.6 MBq·h ± 28.44%), Lesion 3 (4.8 MBq·h ± 17.64%)

## Data Availability

All supporting data are included in the manuscript.

## Abbreviations

Radionuclides/Isotopes	
^224^Ac	Actinium-224
^225^Ac	Actinium-225
^226^Ac	Actinium-226
^227^Ac	Actinium-227
^68^Ga	Gallium-68
^213^Bi	Bismuth-213
^134^Ce/^134^La	Cerium-134/Lanthanum-134
^221^Fr	Francium-221
^222^Rn	Radon-222
^225^Ra	Radium-225
^226^Ra	Radium-226
^232^Th	Thorium-232
^229^Th	Thorium-229
^233^U	Uranium-233
^213^Po	Polonium-213
^209^Tl	Thallium-209
Abbreviations	
AI	Artificial intelligence
BED	Biologically effective dose
CDR	Collimator–detector response
CFs	Calibration factors
CNNs	Convolutional neural networks
CT	Computed tomography
CZT	Cadmium zinc telluride
DL	Deep learning
EBRT	External beam radiotherapy
FDA	Food and Drug Administration
LET	Linear energy transfer
MC	Monte Carlo
MIRD	Medical internal radiation dose
MTD	Maximum tolerated dose
NEMA	National Electrical Manufacturers Association
OSEM	Ordered-subset expectation maximization
PET	Positron emission tomography
PET/CT	Positron emission tomography/computed tomography
PSMA	Prostate-specific membrane antigen
PVEs	Partial-volume effects
QSPECT	Quantitative single-photon emission computed tomography
RBE	Relative biological effectiveness
RPTs	Radiopharmaceutical therapies
SPECT	Single-photon emission computed tomography
SPECT/CT	Single-photon emission computed tomography/computed tomography
TACs	Time–activity curves
TIA	Time-integrated activity
TIAC	Time-integrated activity coefficient
TIACs	Time-integrated activity coefficients
TAT	Targeted alpha therapy
TRT	Targeted radionuclide therapy
VOIs	Volumes of interest
VOI	Volume of interest
VSV	Voxel S-value
